# To what extent does frailty mediate the association between age and the outcomes of brain reperfusion following acute ischemic stroke?

**DOI:** 10.3389/fnagi.2024.1305803

**Published:** 2024-01-25

**Authors:** Luana Aparecida Miranda, Gustavo José Luvizutto, Pedro Augusto Cândido Bessornia, Natalia Eduarda Furlan, Fernanda Cristina Winckler, Natalia Cristina Ferreira, Pedro Tadao Hamamoto Filho, Juli Thomaz de Souza, Luis Cuadrado Martin, Silméia Garcia Zanati Bazan, Gabriel Pinheiro Modolo, Carlos Clayton Macedo de Freitas, Edison Iglesias de Oliveira Vidal, Rodrigo Bazan

**Affiliations:** ^1^Department of Neurology, Psychology, and Psychiatry, Botucatu Medical School, São Paulo State University, Botucatu, São Paulo, Brazil; ^2^Department of Applied Physical Therapy, Federal University of Triângulo Mineiro, Uberaba, Minas Gerais, Brazil; ^3^Department of Internal Medicine, Botucatu Medical School, São Paulo State University, Botucatu, São Paulo, Brazil

**Keywords:** stroke, frailty, thrombolytic therapy, thrombectomy, brain reperfusion

## Abstract

**Objective:**

We evaluated the extent to which frailty mediated the association between age, poor functional outcomes, and mortality after acute ischemic stroke when patients were treated with brain reperfusion (thrombolytic therapy and/or thrombectomy).

**Materials and methods:**

This retrospective cohort study included patients diagnosed with ischemic stroke who had undergone intravenous cerebral reperfusion therapy and/or mechanical thrombectomy. We created a mediation model by analyzing the direct natural effect of an mRS score > 2 and death on age-mediated frailty according to the Frailty Index.

**Results:**

We enrolled 292 patients with acute ischemic stroke who underwent brain reperfusion. Their mean age was 67.7 ± 13.1 years. Ninety days after the stroke ictus, 54 (18.5%) participants died, and 83 (28.4%) lived with moderate to severe disability (2 < mRS < 6). In the mediation analysis of the composite outcome of disability (mRS score > 2) or death, frailty accounted for 28% of the total effect of age. The models used to test for the interaction between age and frailty did not show statistically significant interactions for either outcome, and the addition of the interaction did not significantly change the direct or indirect effects, nor did it improve model fit.

**Conclusion:**

Frailty mediated almost one-third of the effect of age on the composite outcome of disability or death after acute ischemic stroke.

## Introduction

Frailty occurs in approximately one-quarter of all patients (Taylor-Rowan et al., 2019) and is common in older people and is associated with increased mortality, long-term hospitalization, and disability (Mathers et al., 2000; Kwon et al., 2012; Makizako et al., 2015; Miranda et al., 2022). Frailty is defined as a state of heightened risk of adverse health, which is a by-product of the accumulation of age-related health conditions [deficit accumulation model (Rockwood et al., 2002)], and as a biological syndrome characterized by common factors, including weight loss, exhaustion, slow mobility, limited physical activity, and weakness [frailty phenotype model (Fried et al., 2001)].

Sobolewski et al. (2020) suggested that patients who experienced a stroke and are ≥80 years of age can be safely treated with cerebral reperfusion in routine practice. Similarly, Furlan et al. (2021) observed that cerebral reperfusion was a viable treatment for ischemic stroke that did not increase mortality in elderly or very elderly patients. The elderly patients in this Latin American study had worse functional results at hospital discharge and 90 days after stroke. However, the results also reaffirmed the benefits of thrombolytic therapy in stroke patients in this age group. Age was an independent risk factor for disabilities; however, the mortality rate of older patients in this cohort was more strongly related to comorbidities than age.

Additionally, Winovich et al. (2017) observed that frailty was a risk factor for decreased survival and worse recovery after ischemic stroke among people aged ≥65 years. In support of this, Evans et al. (2020) reported that clinical frailty was independently associated with 28-day post-stroke mortality and attenuated improvements in stroke severity following thrombolysis. Furthermore, Miranda et al. (2022) identified frailty as a predictor of acute ischemic stroke outcomes in older adults and reported that it may provide additional prognostic information beyond the determination of stroke severity at hospital admission and facilitate shared decision-making for patients with acute stroke.

However, to the best of our knowledge, the extent to which frailty mediates the effects of age on stroke outcomes has not yet been studied. This information could contribute to our knowledge of the mechanisms underlying the association between aging and poor post-stroke outcomes. Furthermore, from a public health perspective, this would help estimate the stroke-related disability and death that could be avoided through the prevention of frailty among older adults. Therefore, this study aimed to evaluate the extent to which frailty mediates the association among age, poor functional outcomes, and mortality after acute ischemic stroke in patients treated with brain reperfusion (thrombolysis and/or thrombectomy).

## Methods

### Study design, setting, and participants

This was a retrospective cohort study of patients diagnosed with ischemic stroke who had undergone intravenous cerebral reperfusion therapy and/or mechanical thrombectomy. Data were collected from patients who visited the Botucatu Stroke Unit between June 2012 and September 2020. All patients were treated during the acute phase of stroke.

The study included participants who had an ischemic stroke confirmed by neuroimaging, were > 18 years old, had no history of stroke, and had undergone cerebral reperfusion therapy. Individuals were excluded if they presented with other neurological diseases, hemorrhagic stroke confirmed using computed tomography or magnetic resonance imaging, or stroke mimics. All patients were admitted to the hospital [details removed] within the first 48 h of ictus or were referred to the hospital [details removed] in severe cases or in cases of clinical instability that required intensive care support. Patients were followed up at the cerebrovascular disease outpatient clinic 90 days after admission.

### Data collection

All participants were managed at the stroke unit of [details removed] and evaluated by the research team within 72 h of admission. Following the clinical protocol for stroke management at the study institution, the patients were followed up for 90 days through in-person consultations at the cerebrovascular disease outpatient clinic or, if needed, by telephone. Baseline data were collected through direct interviews with patients and their informants, and electronic medical records. This information included sociodemographic data (age, sex, and race), Frailty Index, stroke characteristics (injury site, time from onset of symptoms to hospital arrival, whether patients underwent thrombolytic and/or thrombectomy treatments, and the timing of treatment relative to the ictus), National Institutes of Health Stroke Scale (NIHSS) score at admission, and the patients’ baseline functional status 1 week before the ictus, as classified by the modified Rankin Scale (mRS). We also collected data on the presence of a range of comorbidities (e.g., systemic arterial hypertension, diabetes, dyslipidemia, hypothyroidism, previous myocardial infarction, heart failure, kidney failure, and prior stroke) that were used to construct the Frailty Index (Mitnitski et al., 2001).

### Study variables

#### Exposure variable

In this study, the exposure of interest was patient age, which was treated as a continuous variable in our regression models.

#### Mediator variable

Frailty, as measured by the Frailty Index, was the mediating variable of interest in this study. The Frailty Index is a tool commonly used to assess frailty in epidemiologic studies and is based on the deficit accumulation theory (Rockwood and Mitnitski, 2007). This index is calculated as a ratio of at least 30 diseases, disabilities, symptoms, and abnormal laboratory parameters, which, in this model, are collectively referred to as “deficits.” For example, if an individual has four of 40 such deficits, that person Frailty Index would be 0.1. We created a Frailty Index that included 31 variables (Supplementary appendix 1) following the standard procedure recommended for the creation of such indices (Searle et al., 2008). Each deficit represented by those variables was assigned a score of 1 point when present and 0 otherwise. In our regression analyses, the Frailty Index was treated as a continuous variable as recommended by developers (Mitnitski et al., 2001).

#### Outcome measures

Two outcomes were assessed: overall mortality and disability, which were measured using the modified Rankin Scale (mRS) score 90 days after stroke ictus. The mRS is an ordinal scale that ranges from 0 (no symptoms) to 6 (death). We used modified Rankin Scale (mRS) scores ranging from 3 to 6 to assign a composite outcome of disability or death (Cincura et al., 2009; Baggio et al., 2014).

#### Covariates

The following covariates were used as potential confounders following the disjunctive cause criterion (VanderWeele, 2019): sex, race, stroke severity according to the NIHSS score at admission (recorded as a continuous variable), and smoking status (never smoked, former smoker, or current smoker). Other comorbidities were not included as potential confounders because they had already been used to construct the Frailty Index. The outcomes were not categorized by intravenous thrombolysis (IVT), endovascular therapy (EVT), or mixed because of similar NIHSS scores between the groups.

#### Statistical analyses

We performed descriptive analyses of participants’ baseline characteristics using absolute numbers and proportions for categorical variables. For continuous variables, we used mean and standard deviation (SD) for normally distributed variables or medians and interquartile ranges (IQR) otherwise (Lang and Altman, 2015). For baseline comparisons involving categorical variables, we used the Chi-square test when the expected numbers in each cell of the contingency tables were ≥5, and Fisher’s exact test otherwise. For comparisons comprising continuous variables, we used Welch’s *t*-test for normally distributed variables, and the Wilcoxon Rank Sum test otherwise (Norman and Streiner, 2014).

We performed regression-based causal mediation analysis (Vanderweele, 2015) using the regmedint (Yoshida et al., 2022) R package. In those models, age was the exposure of interest, frailty was the mediator, mRS > 2 and mortality 90 days after the stroke were the outcomes of interest in separate analyses, and sex, race, NIHSS score at admission, and smoking status were the covariates used for adjustment for possible confounding. We also tested for interactions between the exposure (age) and the mediator (frailty) and evaluated multicollinearity by examining variation inflation factors of the regression models (Fox, 2016).

Through the mediation analyses described above we were able to estimate the total effect, the natural direct effect and the natural indirect effect of age on mRS and mortality, as well as the proportion of the total effect of age on those outcomes that was mediated through frailty (Vanderweele, 2015). The total effect corresponds to the effect of changing one unit of the individual’s exposure (age) on the outcome. The natural direct effect corresponds to the effect of changing the individual’s exposure (age) in one unit, while holding the value of the mediator (frailty) constant at the value that would be observed under the specific level of the exposure variable. The natural indirect effect is the effect of changing the mediator value in one unit while holding the exposure the individual’s exposure constant. The proportion mediated is calculated by dividing the natural indirect effect by the total effect and determines the proportion of the total effect of the exposure (age) on the outcome that is mediated by the mediator (frailty).

**Figure 1:**
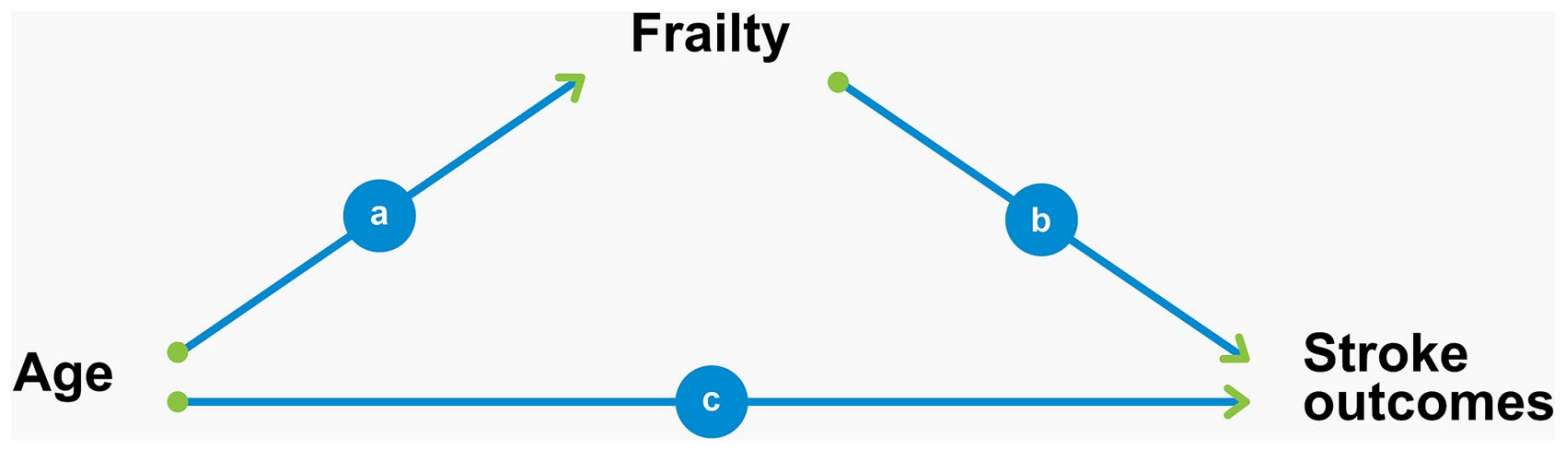
Graphical representation of frailty as a mediator of the association between age and stroke outcomes.

Figure 1 shows the graphical representation of the mediation model and may facilitate the understanding of the mediation analyses performed in this study. The path represented by *c* in that figure corresponds to the natural direct effect described above and stands for the regression coefficient of the age variable calculated by means of a logistic regression of the study outcomes (mRS > 2 and mortality) on age with adjustment for the mediator (frailty) and for confounding due to the other study covariates (sex, race, smoking status, and NIHSS score at admission). The path represented by *b* represents the regression coefficient of the mediator (frailty) in the same logistic regression described above. As to path *a*, it represents the regression coefficient of the linear regression of the mediator (frailty) on the exposure (age) adjusted by the same covariates described previously. The natural indirect effect calculated through the mediation analyses represent the joint effect of paths *a* and *b* together. Finally, the total effect of age on the outcomes represents the joint effect of paths *a*, *b*, and *c*, and is calculated through a logistic regression of the outcomes on age with adjustment for the same covariates but without the mediator.

We did not perform imputation procedures for missing data, which were excluded from the analyses. All statistical analyses were performed using the R software 4.1.2 (R Core Team, 2022). We used a two-tailed alpha level of 0.05 to define statistical significance.

## Results

**Figure 2:**
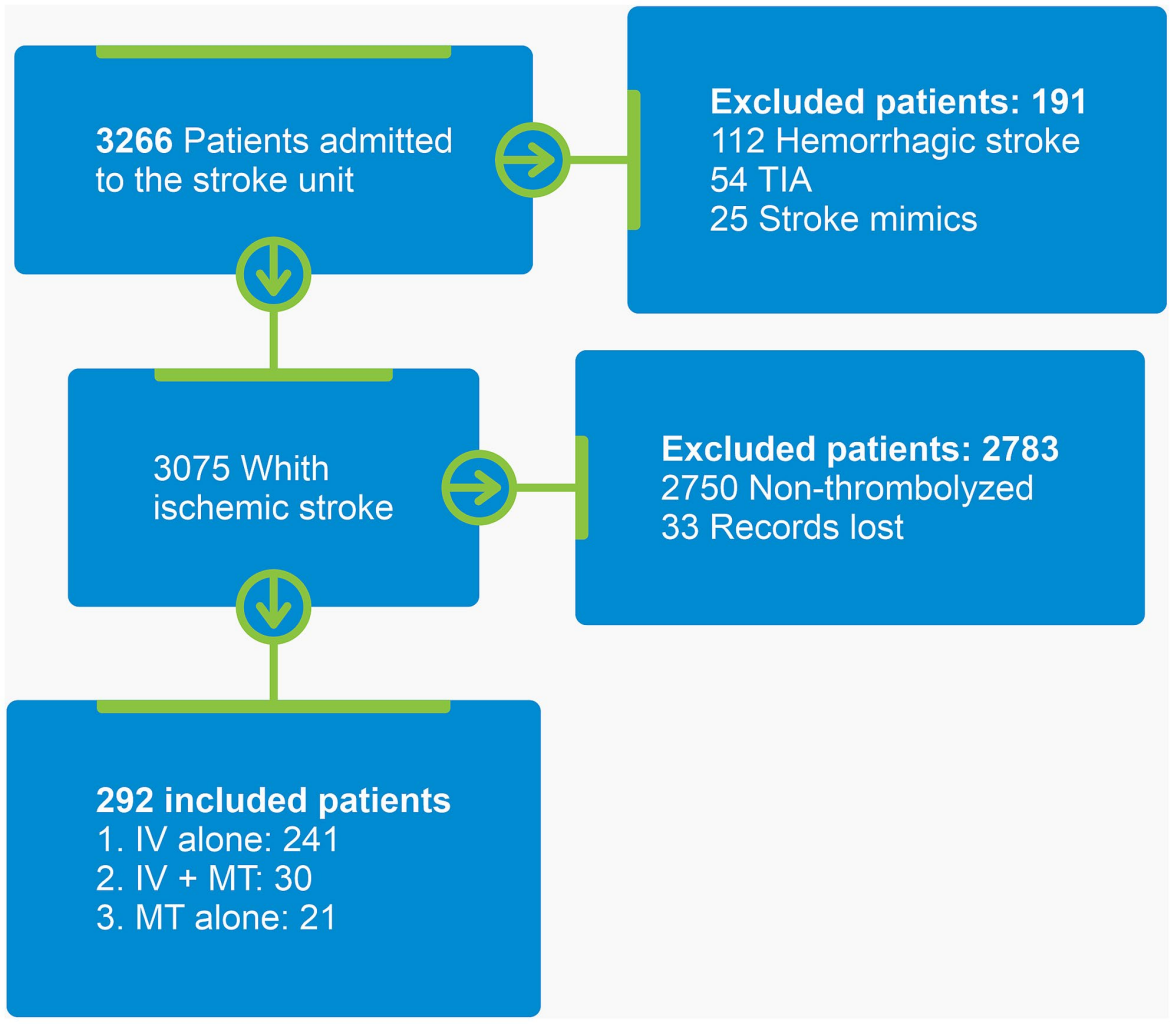
Participant selection flowchart. TIA, Transient ischemic attack; IV, Intravenous thrombolysis; MT, Mechanical thrombectomy.

Figure 2 shows a flowchart of the selection of the participants for this study. A total of 292 participants were included. Their mean age was 67.7 ± 13.1 years, and the majority were white men. Table 1 shows the baseline demographic and clinical characteristics of participants.

**Table 1:** Clinical and demographic characteristics of the study participants.

Characteristic	Proportion (*N* = 292)
**Age, *N* (%)**	
<50 years	26 (8.9)
50–59 years	50 (17.1)
60–69 years	85 (29.1)
70–79 years	69 (23.6)
≥80 years	62 (21.2)
**Sex, *N* (%)**	
Male	165 (56.5)
Female	127 (43.5)
**Race, *N* (%)**	
White	260 (89.0)
Black	32 (11.0)
Frailty index, median (Q1, Q3)	0.10 (0.03, 0.13)
**Modified Rankin Scale, *N* (%)**	
0	202 (69.2)
1	60 (20.5)
2	19 (6.5)
3	8 (2.7)
4	3 (1.0)
Initial NIHSS, median (Q1, Q3)	12.0 (7, 18)
Hypertension, *N* (%)	221 (75.7)
Diabetes, *N* (%)	87 (29.8)
Dyslipidemia, *N* (%)	56 (19.2)
**Smoking, *N* (%)**	
Never smoked	151 (51.7)
Former smoker	56 (19.2)
Current smoker	85 (29.1)
Previous myocardial infarction, *N* (%)	37 (12.7)
Atrial fibrillation, *N* (%)	29 (9.9)
Heart failure, *N* (%)	26 (8.9)
Previous ischemic stroke, *N* (%)	41 (14.0)
Previous acute ischemic attack, *N* (%)	14 (4.8)
Peripheral arterial disease, *N* (%)	6 (2.1)

Q, Quartile; NIHSS, National Institutes of Health Stroke Scale.

**Figure 3:**
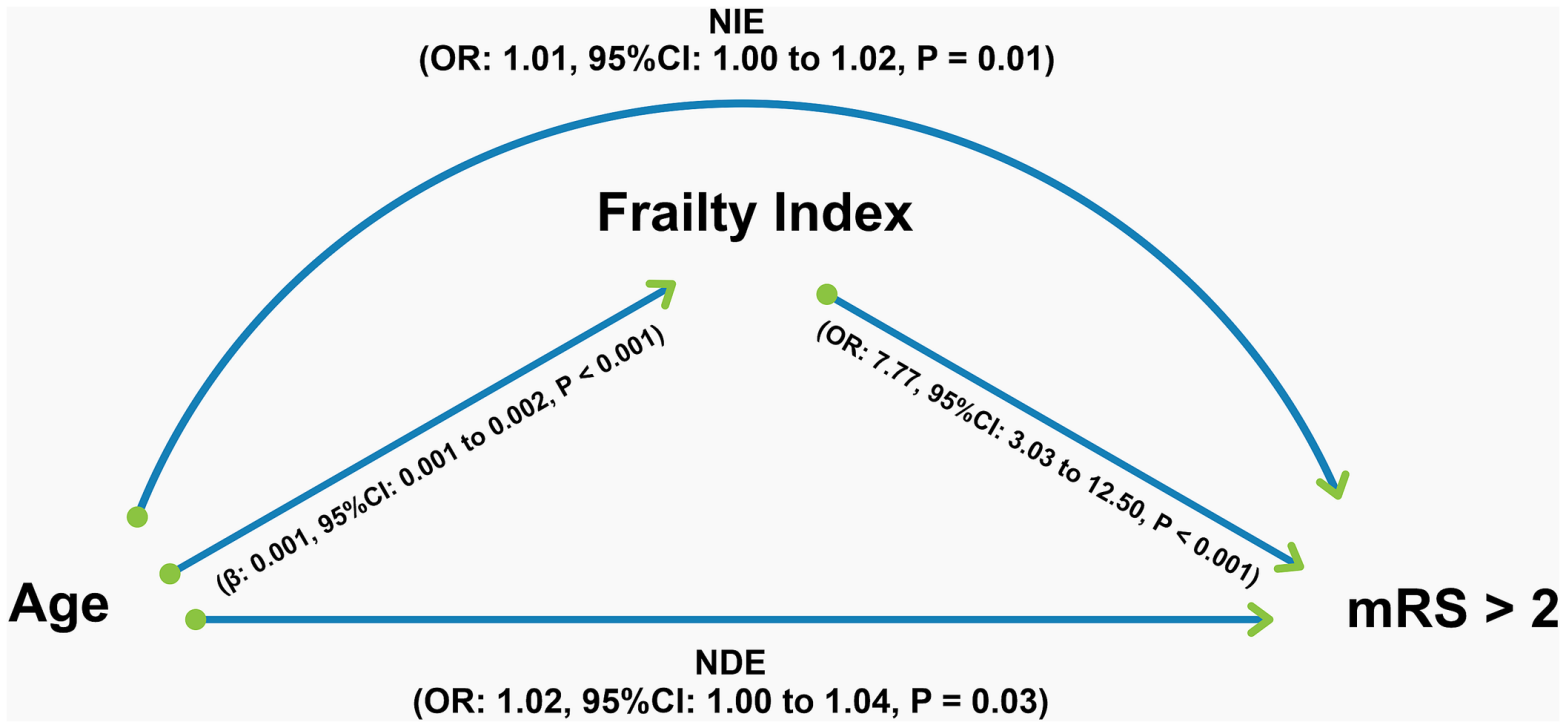
Graphical representation of the results of the mediation analysis for the composite outcome of disability or death (modified Rankin Scale >2) 90 days after the stroke ictus. All regressions underlying the mediation analysis, the coefficients of which are shown in the figure, were adjusted for sex, race, National Institutes of Health Stroke Scale score, and smoking status. NIE, natural indirect effect; NDE: Natural direct effect; OR: Odds ratio; CI: Confidence interval; mRS, modified Rankin Scale.

Ninety days after the stroke ictus, 54 (18.5%) participants died, and 83 (28.4%) lived with moderate to severe disability (2 < mRS < 6). In the mediation analysis of the composite outcome of disability or death (mRS score > 2), frailty accounted for 28% of the total effect of age (Table 2). Importantly, the natural direct effect of age on this outcome remained statistically significant, indicating that the association between age and the composite outcome of disability or death was not fully explained by frailty. Figure 3 shows the results of the regressions underlying the mediation analysis, which revealed that all paths in the mediation diagram were statistically significant and highlighted the strong association between frailty and composite disability/death outcomes. None of the values of the variation inflation factors in our statistical models were above 3. Hence, we are confident that multicollinearity did not bias our results.

**Table 2:** Results of the mediation analysis* for the composite outcome of disability or death (modified Rankin Scale > 2) 90 days after the stroke ictus.

	OR (95% CI)	*p*
Total effect of age on mRS > 2	1.03 (1.02–1.05)	<0.01
NDE of age on mRS > 2	1.02 (1.00–1.04)	0.03
NIE of age on mRS > 2 mediated by frailty	1.01 (1.00–1.02)	0.01
Proportion mediated	27.7% (2.3–53.2%)	0.03

OR, odds ratio; CI: Confidence interval; mRS, modified Rankin Scale; NDE: Natural direct effect; NIE: Natural indirect effect; * Mediation analysis adjusted for sex, race, National Institutes of Health Stroke Scale score, and smoking status.

**Figure 4:**
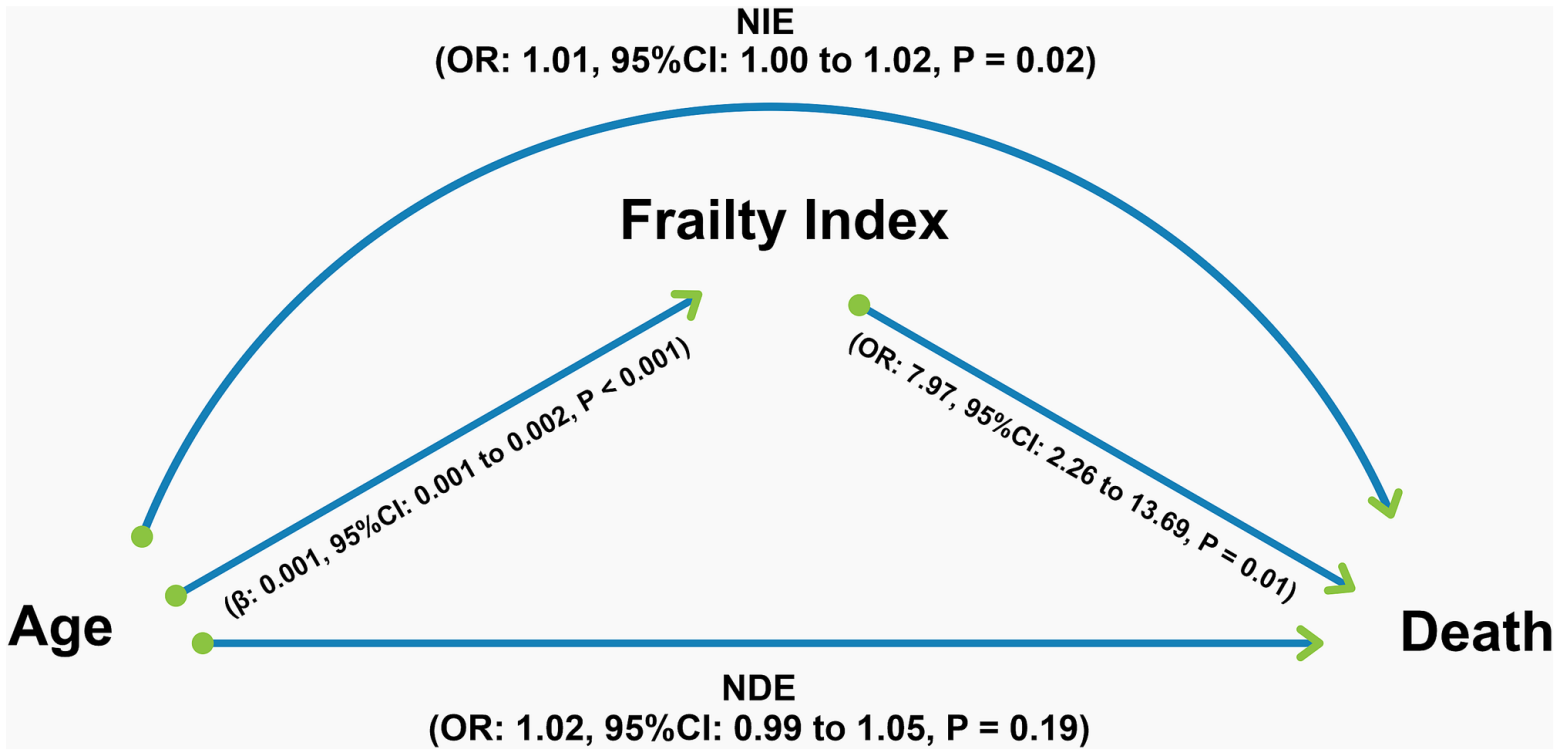
Graphical representation of the results of the mediation analysis for the 90-day mortality outcome. All regressions underlying the mediation analysis, the coefficients of which are shown in the figure, were adjusted for sex, race, National Institutes of Health Stroke Scale score, and smoking status. NIE, Natural indirect effect; NDE, Natural direct effect; OR, Odds ratio; CI, Confidence interval.

Mediation analysis for mortality outcomes showed that 33% of the total effect of age was mediated through frailty, although this was not statistically significant (Table 3). Interestingly, the direct effect of age on mortality was not significant in this model. In contrast, the natural indirect effect, that is, the effect of age on mortality mediated by frailty, remained statistically significant (Figure 4).

**Table 3:** Results of the mediation analysis* for the 90-day mortality outcome.

	OR (95% CI)	*p*
Total effect of age on mRS > 2	1.03 (1.00 to 1.06)	0.04
NDE of age on mRS > 2	1.02 (0.99 to 1.05)	0.19
NIE of age on mRS > 2 mediated by frailty	1.01 (1.00 to 1.02)	0.02
Proportion mediated	32.5% (0 to 72.6%)	0.11

OR, odds ratio; CI: Confidence interval; mRS, modified Rankin Scale; NDE: Natural direct effect; NIE: Natural indirect effect; * Mediation analysis adjusted for sex, race, National Institutes of Health Stroke Scale score, and smoking status.

The models used to test for the interaction between age and frailty did not show statistically significant interactions for either outcome, and the addition of the interaction did not significantly change the direct or indirect effects nor improve the model fit (Vanderweele, 2015).

## Discussion

To our knowledge, this is the first study to evaluate the extent to which frailty mediates the association between age and stroke outcomes. Our results suggest that, if frailty is prevented, approximately 28% of the impact of age on the composite outcome of disability or death can be avoided. Our sample was composed of young and elderly adults, predominantly white and male. The main risk factor was hypertension and diabetes and neurological severity ranged from mild to severe stroke. As is widely known, hypertension and diabetes are among the main causes of stroke (McFarlane et al., 2005), and the frequency of stroke type also differs in ethnic groups; white people have more frequency of large vessels and embolic strokes, while blacks have a higher frequency of small vessels and hemorrhagic strokes (Alter, 1994).

Unfortunately, owing to the limitations of the moderate sample size in our study, the confidence interval of the proportion of the effect of age on the mortality outcome mediated by frailty was wide, and this analysis did not reach statistical significance. Nevertheless, it is noteworthy that the effect of frailty on mortality remained significant, whereas that of age did not. This finding suggests that frailty may have a stronger relationship with the mortality outcome than age itself.

Indeed, for many diseases, frailty is a better predictor of clinical outcomes than age alone. Tan et al. (2022) observed that frail patients who experienced a stroke were more likely to be female and to have more comorbidities than those who were not frail. In addition, the authors showed that frailty was associated with poorer functional outcomes 90 days after endovascular therapy in patients aged ≥70 years. Another study performed at a UK comprehensive stroke center reported that pre-stroke frailty was prevalent in real-world patients eligible for thrombectomy and was an important predictor of poor outcomes. However, age was no longer a predictor of outcomes when adjusted for frailty (Joyce et al., 2022). Although higher frailty status increases the likelihood of poor outcomes and death after endovascular thrombectomy (Pinho et al., 2021; Tiainen et al., 2022), Evans et al. (2020) highlighted that the median NIHSS score improved significantly in non-frail individuals after thrombolysis. Notably, none of these studies evaluated the extent to which frailty mediates the association between age and stroke outcomes.

Our study has a few limitations. First, as discussed, our moderate sample size may not have provided sufficient power to detect a statistically significant proportion of the total effect of age on mortality mediated by frailty. Second, the observational nature of our data does not exclude the possibility of residual confounding involving the causal paths depicted in Figure 1. Importantly, our Frailty Index already included several comorbidities and participants’ baseline functional status, which is the standard for the development of such an index (Searle et al., 2008). Hence, it was not appropriate to include these variables as covariates to adjust for confounding factors in the statistical models. Third, because of the retrospective nature of our data, our Frailty Index was limited to 31 variables and did not include the presence of geriatric syndromes such as falls and incontinence. Nevertheless, the guidelines for the creation of Frailty Indices state that at least 30 variables are required but do not impose the inclusion of any specific items (Rockwood and Mitnitski, 2007; Searle et al., 2008).

In summary, almost one-third of the effect of age on the composite outcome of disability or death after acute ischemic stroke is mediated by frailty. Our study suggests that progress in frailty prevention may have the additional benefit of decreasing the effect of age on negative stroke outcomes in patients undergoing cerebral reperfusion therapy. Furthermore, our findings suggest that beyond age, clinicians should consider their patient’s frailty status when attempting to estimate the prognosis of patients with stroke undergoing reperfusion therapy. Future studies should examine the extent to which frailty, as defined by other diagnostic tools, mediate the association between age and stroke outcomes, and test whether integrating frailty assessments in decision algorithms regarding the deployment of reperfusion therapies leads to improved selection of patients for those therapies.

## Data availability statement

The raw data supporting the conclusions of this article will be made available by the authors, without undue reservation.

## Ethics statement

The studies involving humans were approved by Botucatu Medical School committee. The studies were conducted in accordance with the local legislation and institutional requirements. The participants provided their written informed consent to participate in this study.

## Author contributions

LAM: Conceptualization, Methodology, Project administration, Writing – original draft. GL: Conceptualization, Methodology, Writing – review & editing. PB: Writing – review & editing. NEF: Writing – review & editing. FW: Writing – review & editing. NCF: Writing – review & editing. PH: Writing – review & editing. JdS: Writing – review & editing. LCM: Writing – review & editing. SZ: Writing – review & editing. GP: Writing – review & editing. CF: Writing – review & editing. EV: Formal analysis, Writing – review & editing. RB: Conceptualization, Methodology, Project administration, Supervision, Writing – review & editing.
