# Supplementary material for: To what extent does frailty mediate the association between age and the outcomes of brain reperfusion following acute ischemic stroke?
Source: Front Aging Neurosci. 2024 Jan 25;16:1305803. doi: 10.3389/fnagi.2024.1305803 (PMC10850226; doi:10.3389/fnagi.2024.1305803)
Supplement: Supplementary file 1 [file Table_1.DOCX]

**Appendix 1:** List of variables used for calculation of the Frailty Index

| 1. Arterial hypertension  2. Diabetes mellitus  3. Hypothyroidism  4. Dyslipidemia  5. Glaucoma  6. Generalized anxiety disorder  7. Benign prostatic hyperplasia  8. Alzheimer’s disease  9. Depression  10. Chronic headache  11. COPD or asthma  12. Chronic kidney disease  13. Single kidney  14. Epilepsy  15. Previous myocardial infarction  16. Chronic atrial fibrillation | 17. Paroxysmal atrial fibrillation  18. Chronic heart failure  19. Osteomuscular problems  20. Previous ischemic stroke  21. Previous hemorrhagic stroke  22. Previous transitory ischemic attack  23. Peripheral obstructive arterial disease  24. Syphilis  25. HIV  26. Chagas disease  27. Hepatitis C  28. Anemia  29. Lymphocytopenia  30. Thrombocytopenia  31. Previous modified Rankin Scale > 1 |
| --- | --- |
